# Enhancements to the Rosetta Energy Function Enable Improved Identification of Small Molecules that Inhibit Protein-Protein Interactions

**DOI:** 10.1371/journal.pone.0140359

**Published:** 2015-10-20

**Authors:** Andrea Bazzoli, Simon P. Kelow, John Karanicolas

**Affiliations:** 1 Center for Computational Biology, University of Kansas, 2030 Becker Dr., Lawrence, Kansas, 66045–7534, United States of America; 2 Department of Molecular Biosciences, University of Kansas, 2030 Becker Dr., Lawrence, Kansas, 66045–7534, United States of America; University of Michigan, UNITED STATES

## Abstract

Protein-protein interactions are among today’s most exciting and promising targets for therapeutic intervention. To date, identifying small-molecules that selectively disrupt these interactions has proven particularly challenging for virtual screening tools, since these have typically been optimized to perform well on more “traditional” drug discovery targets. Here, we test the performance of the Rosetta energy function for identifying compounds that inhibit protein interactions, when these active compounds have been hidden amongst pools of “decoys.” Through this virtual screening benchmark, we gauge the effect of two recent enhancements to the functional form of the Rosetta energy function: the new “Talaris” update and the “pwSHO” solvation model. Finally, we conclude by developing and validating a new weight set that maximizes Rosetta’s ability to pick out the active compounds in this test set. Looking collectively over the course of these enhancements, we find a marked improvement in Rosetta’s ability to identify small-molecule inhibitors of protein-protein interactions.

## Introduction

Virtual screening has become an important tool in modern drug discovery, complementing high-throughput biochemical and phenotypic screening to provide small molecules that engage target proteins important in human disease [1]. Already contributions from computer-aided design have helped advance a number of drugs into the clinic [2, 3].

Structure-based (receptor-driven) virtual screening, or “docking,” is comprised of two interrelated tasks: pose generation and ranking. The former involves searching various locations and orientations of each compound in a library, to produce structural models of each potential protein-ligand complex. This search is carried out by optimization of some scoring function, and can include the internal degrees of freedom for the protein and/or for the ligand. Once models have been built for each member of the compound library, these are then ranked to identify those compounds most likely to exhibit the desired biological activity. Ranking may either be carried out using the same scoring function used in pose generation, or by using a more detailed (computationally demanding) approach. By prioritizing a small number of compounds drawn from a much larger library, structure-based screening offers the potential to dramatically reduce the number of compounds that must be tested in the early stages of a drug discovery campaign.

Both tasks are equally critical to successfully selecting active compounds. If an active compound is inappropriately positioned relative to the protein (“mis-docked”), then it is unlikely to achieve a favorable ranking. At the same time, accurately identifying the most promising compounds in the set—without an abundance of false positives—is also essential for harnessing the utility of virtual screening. In this study we focus on the latter step, and ask how recent enhancements to the Rosetta energy function affect its ability to successfully identify active compounds from among large numbers of docked “decoy” compounds.

The Rosetta macromolecular modeling software suite [4] was originally developed as a protein-only tool for structure prediction [5, 6] and design [7], but then grew to allow simulations that can include DNA [8, 9], RNA [10], small molecules [11, 12], non-canonical “peptoid” backbones [13], and even mineral surfaces [14]. Rosetta is supported by a broad community of users studying highly diverse systems, and the underlying energy function is a key contributor to the success of these applications. The Rosetta energy function is typically validated for new applications through two classes of benchmark experiments. The first class focuses on design applications, and includes tests for recapitulation of native sequences [15, 16] and predicting changes in stability/function associated with point mutations [17, 18]. The second class focuses on structure prediction applications, and includes large-scale tests to examine distributions of specific structural features [19] in addition to traditional recapitulation of native rotamers, loops, or complete protein structures [20]. Protocols that introduce new functionality into Rosetta, such as the ability to model and design protein-ligand complexes, are typically evaluated using one or both classes of benchmark [11, 21].

Here, we directly evaluate the performance of the Rosetta energy function in a different type of benchmark: distinguishing known small-molecule inhibitors of protein-protein interactions from large sets of “decoy” compounds. Whereas “traditional” targets for therapeutic intervention include G protein-coupled receptors, ion channels, and various enzymes [22, 23], the role of protein-protein interactions in all aspects of cell growth and development has recently brought extreme attention to this emerging target class [24, 25]. Despite innovative new approaches including fragment-screening [26] and mimetics of protein secondary structural elements [27, 28], biochemical and biophysical methods to identify inhibitors of protein-protein interactions remain more challenging to implement than in campaigns against traditional targets. In principle, the difficulties of “wetlab” approaches in this target space might provide an important opportunity for contributions from virtual screening. In practice, however, computational tools built for screening against traditional targets perform less well when they are deployed at protein interaction sites [29].

**Fig 1:**
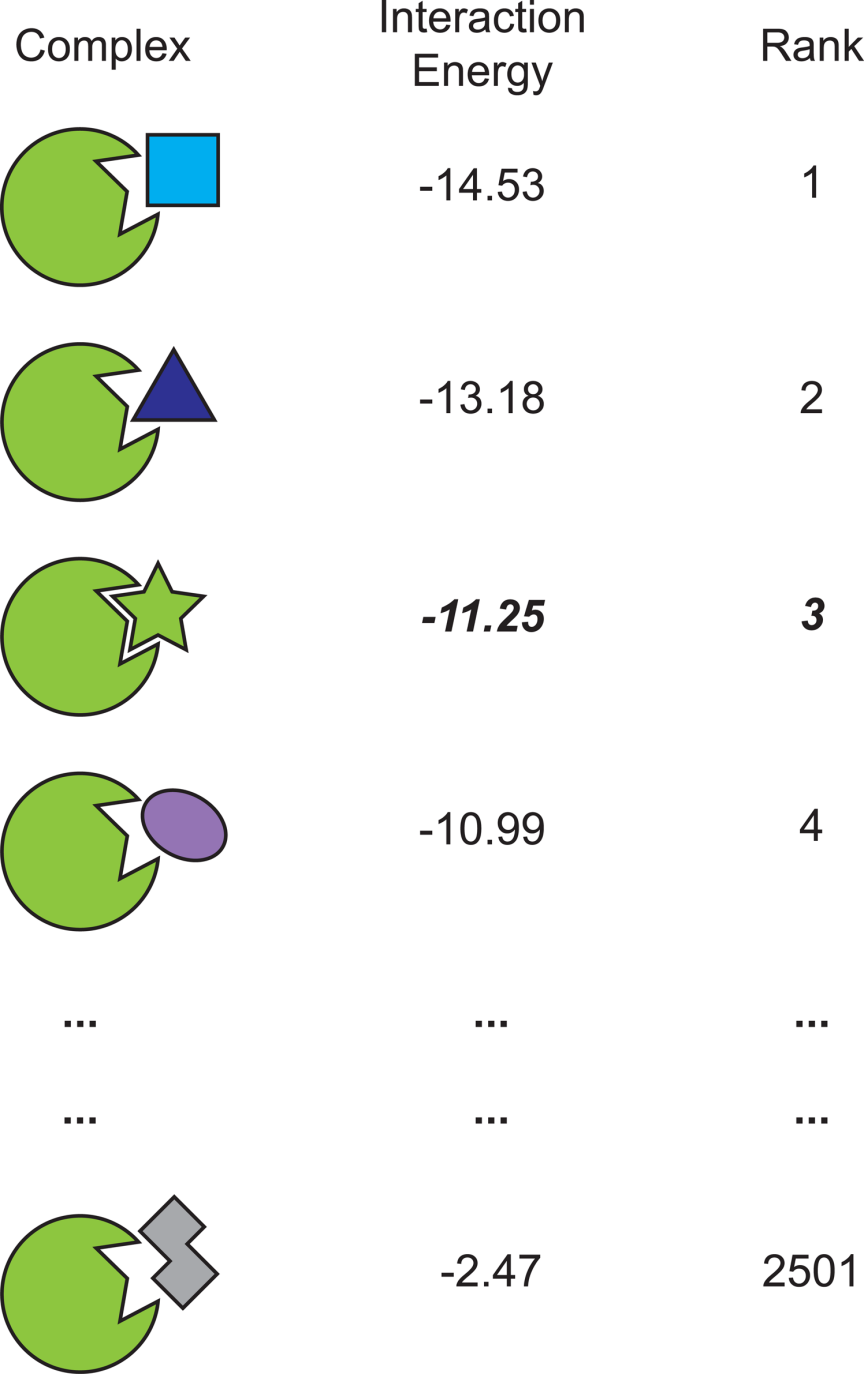
Overview of the virtual screening benchmark. A target protein is provided in complex with a known small-molecule inhibitor acting at this protein interaction site. A collection of 2500 diverse “decoy” ligands have also been docked to this site. The benchmark entails scoring each of the 2501 complexes, and determining the rank of the native ligand relative to the decoy compounds. This experiment carried out for each of 18 non-redundant protein targets (Table 1).

To isolate the Rosetta energy function and examine its performance free from any complications of conformational sampling, our benchmark focuses only on the “ranking” step of virtual screening. For each of 18 non-redundant protein targets with crystal / NMR structures that have been solved in complex with a small-molecule inhibitor (Table 1), we have generated docked models for 2500 diverse “decoy” compounds. By evaluating how Rosetta ranks the active compound for each protein target relative to the decoys (Fig 1), we are able to track changes in performance stemming from updates to the energy function. We will highlight the effects of two very recent additions to Rosetta—Talaris [19, 20, 30] and pwSHO [31]–as well as introduce a new set of weights for the terms in the energy function, to optimize the performance of Rosetta for identifying small-molecule inhibitors of protein-protein interactions.

**Table 1:** Protein targets used in this study. These targets correspond to non-redundant protein-protein interaction sites for which a crystal structure or an NMR structure has been solved in complex with a small-molecule inhibitor. For this study, three protein targets have been removed from our previously reported set [29] (see Methods).

Protein target	PDB ID of ligand-bound structure	Resolution (Å)	R-free
IL-2	1PW6	2.60	0.292
HPV E2	1R6N	2.40	0.266
XIAP-BIR3	1TFT	NMR	NMR
ZipA	1Y2F	2.00	0.228
Bcl-xL	1YSI	NMR	NMR
TNFα	2AZ5	2.10	0.278
HIV-gp41	2KP8	NMR	NMR
BRD4	2YEL	1.65	0.211
S100B	3GK1	2.10	0.257
Grb2-SH2	3IN7	2.00	0.250
SHANK PDZ	3O5N	1.83	0.283
WDR5	3UR4	1.80	0.213
PCNA	3VKX	2.10	0.249
VHL	3ZRC	2.90	0.350
HIV integrase	4E1N	2.00	0.265
Mdm2	4ERF	2.00	0.250
Clathrin	4G55	1.69	0.215
Menin	4GQ4	1.27	0.182

## Methods

### Protein target set

Previously, we described a set of 21 complexes drawn from the 2P2I [32] and TIMBAL [33] databases and supplemented from our own curation of the literature [29]. In cases where a given protein has been separately solved in complex with more than one different ligand, we retained only the structure with the most potent of these ligands. No two proteins in this set are homologs of one another. For the present study, we removed three structures from this set: 1CTR (steric clashes in the crystal structure), 2VC2 (a very large protein), and 1ALW (the geometry of the active site led to poorly docked decoys). The results presented here were obtained using the other 18 complexes (Table 1). Three of these complexes were solved by NMR, and the other fifteen by x-ray crystallography. The resolution of the crystal structures ranged from 1.27 to 2.90 Å, and the R-free from 0.182 to 0.350. Among the complexes for which electron density was available, we visually examined the electron density to ensure that the ligand atomic coordinates were appropriately defined by the density.

### Preparing decoy complexes

The underlying intention is that decoy compounds are expected not to be active against the protein target. To enhance the likelihood that this assumption holds true, we prepared a separate custom set of 2500 “presumed inactive” compounds for each protein target. Drawing from the ZINC database [34, 35], we started by randomly selecting 10,000 compounds with molecular weight between 350 and 550 Da, and hydrophobicity (xlogP) matched to that of the known inhibitor. For each protein target, we then removed from this initial set any compound that was similar in chemical structure to the known inhibitor (2D Fingerprint Tanimoto score [36] ≥ 0.5). To ensure diversity (non-redundancy) of the decoy set, we next removed any compound with similar chemical structure to another compound already included in the set (2D Fingerprint Tanimoto score ≥ 0.8). From the compounds that remained after this filtering, we randomly drew 2500 to form the final decoy set.

To prepare for docking, we used OMEGA [37–39] to generate up to 300 conformers of each decoy compound (using default parameters) [37–39], and defined the binding site for each of the 18 proteins using the bound ligand in the experimentally-determined structure (using the OEDocking “receptor_setup” utility). We then docked each decoy compound to the corresponding protein structure using FRED (with default parameters) [40, 41], and selected the top structure for each compound based on the FRED’s Chemgauss4 score; the resulting structure for each protein-ligand pair was used as a “decoy complex.”

By using FRED (rather than Rosetta) to carry out this docking step, we ensured that any artifacts of the Rosetta energy function would not be inadvertently incorporated into the set of decoy complexes. As part of the benchmark experiments described below, however, each complex (decoy and native) was subjected to an equivalent energy minimization with the particular Rosetta energy function to be used for the subsequent ranking of compounds: in part, this ensures that our evaluation of a given Rosetta energy function is not simply a reflection of how well that Rosetta energy function detects potential artifacts of FRED docking (and uses these to distinguish the native complexes).

### Preparing native complexes

The ranking step in Rosetta (described below) does not currently consider alternate tautomeric states or protonation states of the ligand. The state in the complex may, however, differ from the most likely state existing in solution. Since performance will be evaluated using the correctly-docked pose, it is critical to ensure that the Rosetta calculations are carried out using an appropriate description of the bound ligand. An alternate form of the active ligand would *not* be expected to have activity; thus, these (non-native) forms of the ligand are not suitable for evaluating how well a given method identifies active compounds.

For this reason, we used the Protoss program [42] to obtain the most likely tautomeric state / protonation state for each native ligand, in the context of the protein-ligand complex. By contrast, the most likely tautomeric state / protonation state for the decoy ligands was determined using the QUACPAC toolkit [43], in the absence of the protein. We note that for a real virtual screening application, the native ligand’s tautomeric state / protonation state in the protein environment would not be known *a priori*; those particular active ligands with a configuration that differs in solution versus in the protein environment would simply be missed if the ligand configuration is not adjusted in response to the protein during screening (i.e. false negatives).

We note that this experimental design does disadvantage the decoy ligands relative to the native ligands; we therefore use this benchmark to track *differences* in performance—and not absolute performance—of various scoring functions.

### Availability of the benchmark set

The entire benchmark set is available as (S1 Benchmark). For each target protein, this includes the protein itself, the native ligand (in the experimentally-determined pose and in the FRED-docked pose), and the decoy ligands (in the FRED-docked pose). The native ligand is provided with both the Protoss and the QUACPAC tautomeric state / protonation state.

### Ranking complexes using Rosetta

Prior to scoring each complex, we used the “molfile-to-params” application [12] to assign atomic chemical properties and generate parameter definitions for each ligand. We then performed gradient-based energy minimization of the complex, using the same Rosetta energy function that would later be used to score and rank each complex. All internal dihedral angles of the ligand and of the protein (backbone and sidechains) were included as degrees of freedom in this step, along with the ligand’s position and orientation relative to the protein.

After minimization, the protein-ligand interaction energy was evaluated using the desired energy function. The rank of the active compound’s interaction energy was determined relative to those of the decoy compounds, as shown in Fig 1, from 1 (best) to 2501 (worst). We have made available the ranking of the active compound for each protein target under each energy function in this study (S1 Dataset); these data comprise the results described below.

### Statistical analysis

To compare the performance of two different energy functions, we considered—for each of the 18 proteins in our test set—which energy function positioned the native compound at a more favorable rank relative to the decoys, and assigned that energy function a “win” for the protein target; we then counted the total number of “wins” from each of the two energy functions. To avoid noise from very small differences in ranking, we excluded from the comparison any protein target for which both rankings were within 10% of one another.

To determine the statistical significance of performance differences exhibited by two different energy functions, we started from the null hypothesis that both energy functions performed equally well. Our comparisons are between two sets of paired samples (i.e. the protein targets), and the rankings may not be normally distributed; for these comparisons we therefore applied the (non-parametric) Wilcoxon Signed-Rank test. All comparisons were applied to the logarithms (base10) of the rankings. We used the implementation of this test in the R statistical computing environment [44], invoked as follows:

wilcox.test(mydata$scorefxn_A, mydata$scorefxn_B, paired = TRUE, alternative = "greater")

Because this test uses the *rank values* in the two energy-function sets, it has the advantage of taking into account the *magnitude* by which an energy function “wins” a given protein target; this would not be the case if we were to simply compare the *number* of “wins” by each energy function.

In light of the chronological order by which the improvements to the energy function occurred, we had a prior expectation of which version would outperform the other; for this reason, throughout this study we conducted our analyses using one-tailed tests.

## Results

### Performance of the 2008–2013 Rosetta energy function

As a starting point for further comparisons, we began with the default Rosetta energy function from early 2013, “score12”. Prior to recent improvements, this version of the energy function had remained essentially unchanged over a period of ten years [6]. For each of the 18 protein targets, we used this energy function to score the 2501 complexes and evaluate the rank of the native inhibitor (Fig 1). As an objective point of comparison, we also carried out the same task using the most recent scoring function provided with FRED, Chemgauss4 [40, 41].

**Fig 2:**
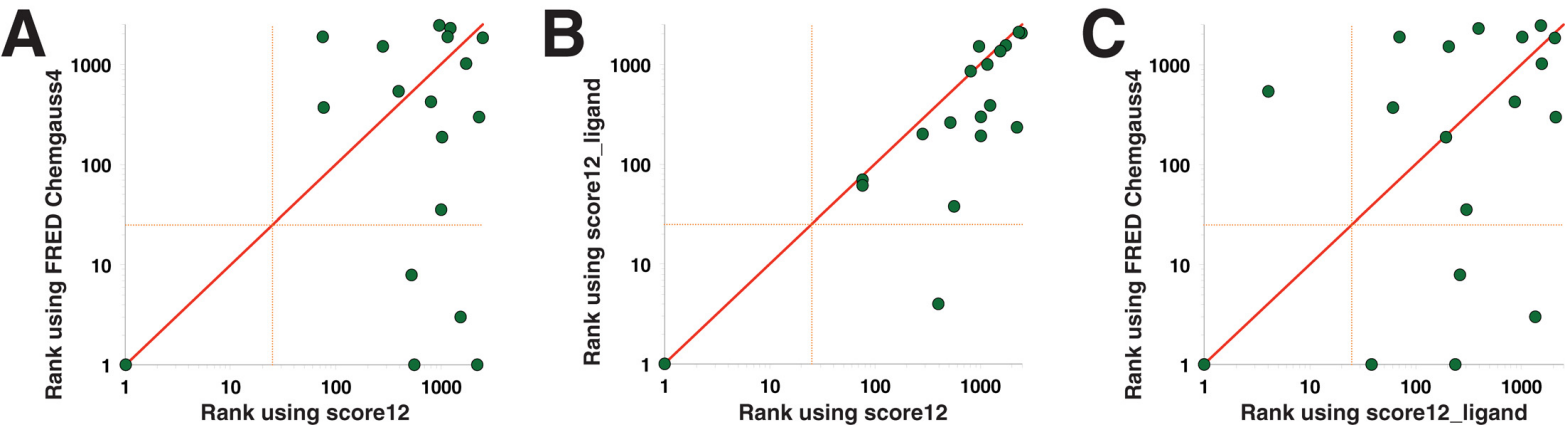
Baseline performance of the Rosetta energy function prior to recent enhancements. Each plot compares the performance of two different scoring functions for identifying the active compounds in our virtual screening benchmark. Each of the 18 protein targets corresponds to a single point (*green dots*); the rank of the active compound (relative to 2500 diverse “decoy” compounds) by each scoring function is indicated. The orange dotted line indicates a ranking of 25, corresponding to the top 1% of the decoy set. **(A)** FRED’s Chemgauss4 energy function outperforms Rosetta’s original energy function intended for protein-only systems, score12, but not at a statistically significant threshold (p = 0.085). **(B)** The variant of the score12 energy function that was developed specifically for modeling protein-ligand interactions, score12_ligand, offers improved performance over score12 (p = 0.001). **(C)** FRED’s Chemgauss4 energy function performs at a similar level as score12_ligand (p = 0.239). All p-values are calculated by applying the Wilcoxon Signed-Rank test to the logs of the ranks (see Methods).

In Fig 2A we compare the performance of the Rosetta score12 energy function to that of FRED’s Chemgauss4. We find that Chemgauss4 outperforms score12 in 10 of the 18 testcases (*points below the diagonal*). Nonetheless, it is important to note that in many of the cases for which score12 “wins”, neither method performs particularly well—in 5 of these 7 cases the active compound is ranked outside the top 100 by both methods. In contrast, many of Chemgauss4’s “wins” correspond to very good rankings of the active compound: in five cases, the active compound is ranked by Chemgauss4 in the top 25 (corresponding to the top 1% of the set), and in three cases the active compound is ranked first overall—ahead of *every* decoy complex. Both scoring functions have the same (optimal) performance on target 3ZRC, the only one ranked first overall by score12.

It is clearly important to consider the magnitude of the differences in the rankings when comparing two scoring functions, and not just the number of protein targets at which each method “wins”. To do so, we compared these scoring functions using the Wilcoxon Signed-Rank test, applied to the difference in the logs of the rankings (see Methods). Based on this test, we cannot quite conclude statistical significance for the observation that FRED’s Chemgauss4 is more effective at ranking the known inhibitors (p = 0.085). Here, the relatively small number of available testcases limits the statistical significance of the comparison; this is an unfortunate consequence of the overall dearth of small-molecule inhibitors of protein-protein interactions reported in the literature to date (and correspondingly, the lack of available crystal structures). Even when the p-value indicates that the difference in performance between the two scoring functions is not statistically significant (p ≥ 0.05), however, it can still provide a useful way to quantify the performance differences.

This first experiment made use of score12, a “standard” Rosetta energy function intended to be used in protein-only simulations. For studies involving small molecules, an alternative energy function is typically preferred [11]: this one shares most energy terms with score12, but combines them in a different linear combination (the so-called “ligand” weight set). Unsurprisingly, we find that this “score12_ligand” energy function outperforms score12 in identifying the native ligands in our benchmark (Fig 2B): performance is improved for 13 protein targets, and worse for only 1 target (for the remaining 4 targets, the rankings from the two energy functions are within 10% of one another and are thus considered “ties”). This improvement is further reflected in the fact that the active compound is now ranked in the top 1% in two cases. Applying the Wilcoxon Signed-Rank test, we conclude that the improvement in ranking associated with score12_ligand is indeed statistically significant (p = 0.001).

The improvement from score12 to score12_ligand is also evident when score12_ligand is compared to FRED’s Chemgauss4 (Fig 2C). FRED’s Chemgauss4 “wins” only 2 targets more than score12_ligand (9 vs. 7), and the similar performance of these two scoring functions is also reflected in the Wilcoxon Signed-Rank test (p = 0.239).

### Talaris and pwSHO

Talaris, the updated energy function mentioned above, became available to the Rosetta community in June 2013. This major update contained multiple improvements throughout the energy function [19, 20, 30], most notably including: an updated functional form for the hydrogen bond term, an explicit Coulombic electrostatic term (“fa_elec”) to replace the previous low-resolution, knowledge-based term (“fa_pair”), adjusted parameters in the Lennard-Jones and solvation terms, and new bicubic interpolation and rotamer library for the knowledge-based terms. We anticipated that the improvements in the hydrogen bond term and the electrostatic term, in particular, would prove particularly important in this virtual screening benchmark.

**Fig 3:**
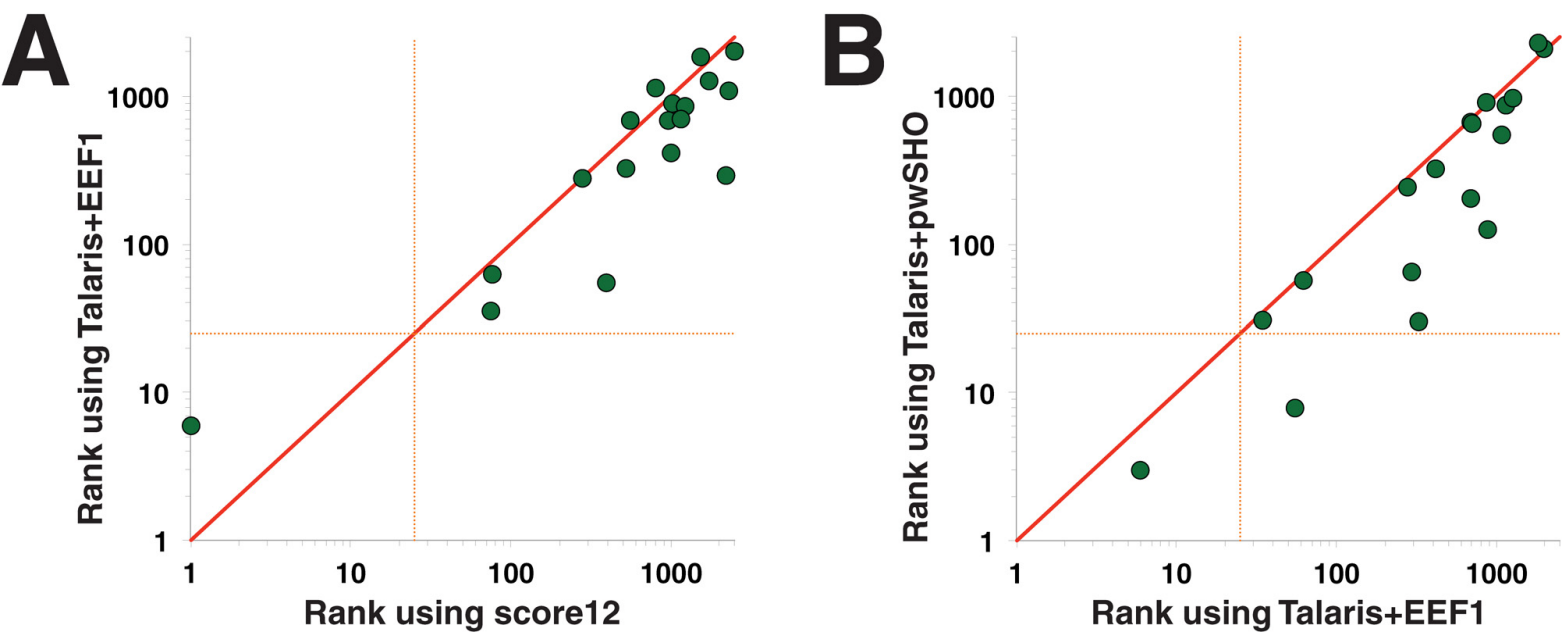
Recent enhancements to the functional form of the Rosetta energy function enable improved performance. **(A)** Rosetta’s “Talaris” energy function [19, 20, 30] includes updates to the functional form of the hydrogen bond term and of the electrostatic term. These changes lead to improved performance relative to score12, at a statistically significant level (p = 0.013). **(B)** Replacing Rosetta’s default model of polar solvation, EEF1, with a newly developed model, pwSHO [31], leads to further improvement at a statistically significant level (p = 0.0004).

Ligand-specific weights have not yet been developed for Talaris; accordingly, we compared the performance of the default Talaris to that of the default score12, since both energy functions are intended primarily for simulations of proteins alone. We find that the use of Talaris improves performance for 13 protein targets relative to score12, with decreased performance in only 4 cases (performance was unchanged for one target) (Fig 3A). This difference was associated with marked improvement in the overall rankings as well, as determined from the Wilcoxon Signed-Rank test (p = 0.013).

An ancillary utility of this benchmark is that the detailed energetic contributions for individual complexes can help rationalize why a given energy function outperforms another. In particular, this insight can be gained from complexes—either native or decoy—that are ranked favorably by one energy function, and less favorably by the other. To do so we compared score12 and Talaris using the same (non-minimized) structures of the decoy and native complexes. This choice allowed us to directly link differences in ranking to differences in the evaluation of energy components—a connection much more difficult to draw in the case of minimized decoys, since the conformation changes slightly in response to the energy function. Two examples are provided by the decoy complexes shown in S1 Fig: their very favorable ranking by score12 but not by Talaris can be explained in terms of score12’s less-refined treatment of solvation, electrostatics, and hydrogen bonding.

Burial of polar groups is penalized by Rosetta’s solvation model; by default this has been EEF1 [45]. In the context of this benchmark we examined the effect of replacing this model of polar solvation with a newly developed model, pwSHO [31], that was introduced to the Rosetta community in July 2014. Whereas EEF1 determines the desolvation penalty by simply integrating the occluded volume around a polar group, pwSHO additionally considers the energetic “value” of the water that has been displaced from this volume. For example, displacing water that would otherwise make a strong hydrogen bond to the polar group of interest is penalized more strongly than displacing water that would not. In its current implementation pwSHO is on the same energy scale as EEF1, such that it can be used to replace EEF1 without any further re-weighting of the energy function. Since pwSHO describes only the polar part of desolvation, the non-polar part of EEF1 (energetics of burying hydrophobic groups) is retained when using pwSHO. In separate simulations using pwSHO in place of EEF1, we observe fewer output structures containing buried “unsatisfied” polar groups (i.e., polar atoms sequestered away from the solvent but not participating in compensatory intramolecular/intermolecular hydrogen bonds). Given the importance of solvation for inhibitors of protein-protein interaction (due to their characteristic shallow binding pockets), we anticipated that pwSHO would also assist in identifying the active compounds in this experiment.

Upon replacing EEF1 with pwSHO in the Rosetta energy function, we find improved performance for 12 of the 13 protein targets exhibiting differences in rankings (Fig 3B), with notable differences in the ranking of the active compounds by the Wilcoxon Signed-Rank test (p = 0.0004). Thus, modeling protein-water interactions through pwSHO leads to accumulation of higher energetic penalties on decoy atoms for their unfavorable desolvation (S2 Fig), which in turn proves beneficial to the discrimination of known inhibitors from decoy ligands.

Collectively, these results suggest that the enhancements to the Rosetta energy function from Talaris and pwSHO together provide improved performance relative to score12. However, it must still be recalled that the performance of score12_ligand was far superior to that of score12, since the former was parameterized specifically for protein-ligand interactions.

### Optimal weights for identifying small-molecule inhibitors of protein-protein interactions

In a previous benchmark experiment, we found that virtual screening by FRED was exceptionally effective for traditional drug targets (proteins naturally evolved to bind small molecules), but much less so for identifying inhibitors of protein-protein interactions [29]. Much like FRED’s Chemgauss4 scoring function, Rosetta’s score12_ligand weights [11] were optimized for performance on a broad variety of protein-ligand interactions rather than for a particular class of interaction. Further, others have reported that performance can be enhanced by using weights optimized for a particular class of protein-ligand interaction [46]. Accordingly, to investigate the extent that performance might be improved under the current functional form of the Rosetta energy function (with Talaris and pwSHO), we next built a set of weights tailored to small-molecule inhibitors of protein interactions.

There are seven component energy terms in Rosetta that contribute to the total intermolecular energy of a protein-ligand complex:

fa_atr (attractive portion of the Lennard-Jones potential)
fa_rep (repulsive portion of the Lennard-Jones potential)
lk_nonpolar (the hydrophobic part of EEF1)
fa_elec (electrostatics with a distance dependent dielectric)
hb_bb_sc (hydrogen bonds between the ligand and the protein backbone)
hb_sc (hydrogen bonds between the ligand and the protein sidechains)
occ_sol_fitted (pwSHO)

These terms are combined in the energy function as follows:

$${\text{E}}_{\text{interface}}=\text{C}1{*E}_{\text{fa}\_\text{atr}}+\text{C}2{*E}_{\text{fa}\_\text{rep}}+\text{C}3{*E}_{\text{lk}\_\text{nonpolar}}+\text{C}4{*E}_{\text{fa}\_\text{elec}}+\text{C}5{*E}_{\text{hb}\_\text{bb}\_\text{sc}}+\text{C}6{*E}_{\text{hb}\_\text{sc}}+\text{C}7{*E}_{\text{occ}\_\text{sol}\_\text{fitted}}$$

We fixed the weight of “fa_atr” (C1) at 0.8, its value in the Talaris 2013 energy function. We also fixed the weight of “lk_nonpolar” (C3) at 0, after preliminary runs of the optimization procedure (described in the next paragraph) converged to negative C3 values; this term favors close packing of hydrophobic groups, and is thus somewhat redundant with “fa_atr”. Developing a new weight set, then, required determining the optimal values for C2/C4/C5/C6/C7.

For this purpose, we first extracted the component energies for each native and decoy complex in our benchmark. We then used Nelder-Mead simplex optimization, as implemented in the C++ GSL multidimensional minimization library (http://www.gnu.org/s/gsl/), to optimize the values of C2/C4/C5/C6/C7; the objective function was defined to be the sum of the logs of the ranks of the native complexes relative to the corresponding decoys. Thus, each step of this simplex optimization entailed updating 45,018 energies (18 protein targets × 2501 complexes for each) using a new set of weights. Because we had pre-computed the component energies for each of these 45,018 complexes, however, updating the total energy for each complex and re-evaluating the rank of the native ligand for each protein target was trivial (and did not require calling Rosetta); thus, the optimization could be completed in seconds. Given the stochastic nature of the Nelder-Mead simplex algorithm, we ran 5 independent optimizations, then selected the set of weights that yielded the minimum value for the objective function.

The resulting weight set, trained on all 18 protein targets, is now available in Rosetta distributions alongside the other standard weight sets, with the name “PPI_discrimination.wts”. Examination of these weights (S2 Dataset) reveals that the “hb_bb_sc” energy term is strongly upweighted (by a factor of 3.5), suggesting that favorable hydrogen bonding with the protein backbone is a distinctive feature that helps identification of small-molecule inhibitors of protein-protein interactions. The electrostatic term (“fa_elec”) and the pwSHO term (“occ_sol_fitted”) are also upweighted (by a factor of 2.35 and 1.55, respectively). The weights for the “hb_sc” term and the “fa_rep” term are decreased (each by a factor of 0.8), though this may arise from high variability of these two weights across training sets (S2 Dataset). Taken together, the optimized weights highlight the importance of polar contacts in complexes of small molecules that employ shallow binding modes, such as those acting at protein interaction sites. It is important to note that these new weights are intended to be used in virtual screening tasks that entail scoring and ranking of (minimized) static structures; these weights are *not* intended to be used in simulations (including energy minimization) that alter the structure of the protein-ligand complex.

To fairly evaluate the performance of this new energy function relative to the others we have considered so far, we used leave-one-out cross validation. For a given protein target, we first developed a unique weight set by training on the other 17 protein targets; we then applied this “custom” weight set to determine the rank of the native complex for the protein target of interest. We repeated this procedure for each of the 18 protein targets, and used the rankings obtained in this manner for the comparisons below.

**Fig 4:**
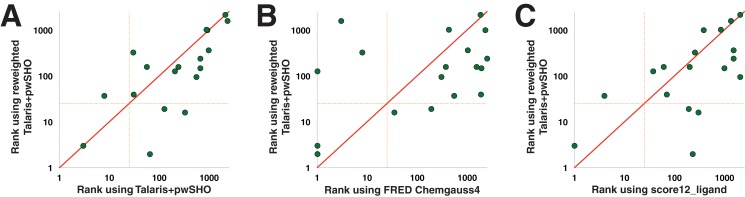
New weights for identifying small-molecule inhibitors of protein-protein interactions. Using the Talaris+pwSHO functional form of the energy function, we identified new weights to optimize Rosetta’s ability to distinguish true inhibitors from decoy compounds. The results presented here were obtained by leave-one-out cross validation of the weights over the benchmark set of 18 non-redundant protein targets. **(A)** Reweighting of the Talaris+pwSHO energy function leads to improved performance, though not at a statistically significant level (p = 0.078). **(B)** This new reweighted energy function provides comparable performance as FRED’s Chemgauss4 energy function (p = 0.221). **(C)** The new reweighted energy function slightly outperforms score12_ligand, though again not to a degree attaining statistical significance (p = 0.123).

First, we compared the performance of these new weights to that of the Talaris+pwSHO function with standard weights (Fig 4A). We observe that the new weight set “wins” 10 targets, whereas standard Talaris+pwSHO “wins” only 5. While this improvement in performance is not statistically significant (p = 0.078), we find that in 4 cases the active ligand is now included in the top 1% of the compound library, and in 7 cases it is ranked among the top 100 compounds. Comparison to FRED’s Chemgauss4 (Fig 4B) shows that this new Rosetta weight set performs comparably (p = 0.221). Finally, we find that this new weight set also slightly outperforms score12_ligand (Fig 4C), albeit not by a statistically significant margin (p = 0.123). We anticipate that the improvements of this new energy function derive from a combination of Talaris, pwSHO, and weights that were developed specifically for identifying inhibitors of protein-protein interactions.

## Discussion

In this study we evaluated the performance of the Rosetta energy function for an important and timely virtual screening application: selecting active compounds that will inhibit protein interaction sites, from among large libraries. As we noted earlier, this is a particularly challenging task compared to virtual screening against “traditional” drug targets [29]. This class of protein-ligand interactions is mediated by shallower binding modes than those observed in “traditional” complexes, and accordingly sterics provide less information to guide identification of active compounds. Instead, polar contacts are of increased importance, and long-standing challenges in accurately describing the energetics of polar interactions may thus underlie difficulties in computationally screening against protein interaction sites. Further, sub-optimal performance may also result from the use of tools parameterized for “traditional” targets.

In the course of the studies presented here, we have developed a new set of weights for the Rosetta energy function that is specifically designed for identifying candidate inhibitors to disrupt protein interaction sites. Critically, this weight set is *not* intended for carrying out simulations that alter the structure of protein-ligand complexes, but rather is focused solely on the final “re-ranking” step that determines which compounds will be advanced for testing in biochemical or cell-based assays.

Virtual screening can be split—both conceptually and pragmatically–into two separate tasks: docking each member of a compound library, and then re-ranking the docked complexes. Here, we focus exclusively on the latter task and have developed a benchmark experiment specifically designed to evaluate performance for this task. In doing so, however, we note that our benchmark does differ in certain key aspects from the scenario of a “true” virtual screen.

First, our benchmark makes use of FRED-docked decoy complexes, but experimentally-derived native complexes (with subsequent minimization of both). In a real virtual screening scenario, of course, *all* of the complexes come from docking. Indeed, we find that using FRED-docked native complexes, instead of the experimentally-derived complexes, leads to diminished performance in this benchmark (S3 Fig). While this is a more realistic experiment, it does not clearly decouple the performance of the docking task from the re-ranking task. An incorrectly docked pose should *not* score favorably, even if the compound happens to be a true inhibitor. Failure to identify the correct pose is a deficiency of the docking step, and thus should not influence the results of a benchmark for the re-ranking task.

Our benchmark also makes use of the tautomeric state / protonation state for the known ligand that best fits the protein environment in the correctly-docked pose, as identified by Protoss. Again this assumes knowledge that would not be available in a true virtual screening scenario, and again failure to provide this knowledge (in this case, relying on QUACPAC to provide the configuration, without insight from the protein environment) leads to diminished performance (S4 Fig). In this case too though, a docked pose in which the ligand configuration is not matched to the protein environment should not score favorably; thus, it is most helpful to utilize a high-resolution benchmark that stresses identification of the known inhibitor only when this compound can be selected “for the right reason.”

Finally, we note that each of the complexes in our benchmark are minimized using the same scoring function that is used to ranking the complexes. Carrying out this calculation for each compound in a very large library is often impractical, and thus minimization is typically only used for the top-scoring compounds after docking (if at all). In this case the minimization step does not provide extra knowledge that would not be available in a realistic scenario, however, but rather the opposite: FRED-docked decoys contain more steric clashes than the corresponding experimentally-derived complexes, and as a result it becomes nearly trivial to pick out the (non-docked) active complex (S5 Fig). Though minimization of a complete library may not be practical for a large-scale virtual screen, it was a necessary component of this benchmark in order to ensure that the decoy compounds were not unfairly disadvantaged.

Using the benchmark we describe here, the performance of Rosetta for this task can be directly tracked across several recent enhancements to the energy function; all the comparisons described in the course of this study are summarized in Table 2. We note that this is still an exceedingly challenging benchmark, as highlighted by the relatively poor performance of score12 at the outset of our study. Hence, any substantial improvement in rank may indicate that the underlying modification to the energy function is worth pursuing further, even if the absolute rank is still unimpressive. However, given that the number of compounds advanced to experimental validation rarely exceeds a few dozen, improvements at low rank (i.e. among the most favorably scored compounds) should be valued more than improvements at high rank. Together, these two considerations motivate our decision to apply the Wilcoxon Signed-Rank test to the complete set of compounds (1–2501), and also to compare ranks in terms of their logarithms. It is thus exciting and encouraging that Rosetta’s ability to identify the active compounds in this benchmark was improved to the extent observed in this study—ultimately bringing it to a level of performance similar to that of FRED’s Chemgauss4 scoring function.

**Table 2:** Summary of the comparisons made in this study. Among the 18 protein targets used as testcases, those for which the rankings by both scoring functions were within 10% of one another were considered to be “ties”. The reported p-values were calculated by applying the Wilcoxon Signed-Rank test to the difference in the log of the rankings, over all testcases. This (non-parametric) statistical test has the advantage that the degree to which a given method “wins” each testcase—and not just the number of “wins”—is taken into account.

Comparison	Fraction of targets with improved performance	p-value (Wilcoxon Signed-Rank test)
FRED Chemgauss4 outperforms score12	10 / 17	0.085
score12_ligand outperforms score12	13 /14	0.001
FRED Chemgauss4 outperforms score12_ligand	9 / 16	0.239
Talaris outperforms score12	13 / 17	0.013
Talaris+pwSHO outperforms Talaris+EEF1	12 / 13	0.0004
Reweighted Talaris+pwSHO outperforms original Talaris+pwSHO	10 / 15	0.078
Reweighted Talaris+pwSHO outperforms FRED Chemgauss4	11 / 18	0.221
Reweighted Talaris+pwSHO outperforms score12_ligand	9 / 17	0.123

Nonetheless, even with these enhancements both FRED's Chemgauss4 and Rosetta fail to rank the known inhibitor in the top 1% of compounds for most targets, confirming that identifying small-molecule inhibitors of protein-protein interactions remains a challenging task. Given the complexity of this task, it also remains difficult to ascertain precisely why current methods fall short. It is noteworthy, though, that considerable orthogonality remains between Chemgauss4 and Rosetta. If both methods represented slight variations of the same underlying approach, one would expect them struggle/excel on the same targets as one another. Clearly, however, this is not the case: among the five targets for which Chemgauss4 ranked the known inhibitor in the top 1%, Rosetta performed comparably in only two cases, and in one case Rosetta failed even to rank the known inhibitor in the top 50%; conversely, Rosetta had more “wins” in the same experiment (Fig 4B). These differences imply that Chemgauss4 recognizes important energetic features of the native interaction that are missed by Rosetta, and vice versa; we are therefore optimistic that detailed examination of complexes for which the two cannot agree (S6 Fig) will reveal further avenues for improving the both energy functions.

## Supporting Information

S1 Benchmark All files needed to run this benchmark are provided. For each target protein, this includes the protein itself, the native ligand (in the experimentally-determined pose and in the FRED-docked pose), and the decoy ligands (in the FRED-docked pose). The native ligand is provided with both the Protoss and the QUACPAC tautomeric state / protonation state.
(GZ) Click here for additional data file.

S1 Dataset Complete ranking data. Ranking of the active compound relative to the decoys is provided for each protein target, under each of the energy functions tested in this study; these data were used in generating the figures presented in this study.
(XLSX)

S2 Dataset Standard weights and optimized weights for the Talaris+pwSHO energy function. The weights optimized on the entire benchmark set are those deposited in “PPI_discrimination.wts”. The mean and standard deviation of the weights optimized during leave-one-out (over the 18 rounds of the procedure, where each round optimized on a different subset of 17 targets) are also reported.
(XLSX)

S1 Fig Rationalization of decoys that rank favorably with score12, but less so with Talaris. To appropriately compare between the two energy functions, the same conformation was scored using both energy functions (i.e. without minimization). Both images were produced using PyMOL [47], with the protein represented in spheres and the ligand represented in sticks. (**A)** The protein PDB ID is 4GQ4, the ligand ZINC ID is ZINC01019586. This decoy complex is ranked 10th by score12 and 34th by Talaris, with protein-ligand interface energies of -10.522 and -8.913 Rosetta Energy Units (REUs), respectively. Seventy-three percent of the difference in interface energy is due to the solvation component of the interface energy, which is +8.397 under score12 and +9.564 under Talaris. The change reflects the increase from 0.65 in score12 to 0.75 in Talaris in the weight that solvation energy has in the computation of total energy; the unweighted solvation components are indeed very similar: +12.918 for score12 and +12.752 for Talaris. The electrostatic component contributes 20% of the difference in interface energy, because the score12 “fa_pair” component is 0 REUs, whereas the Talaris “fa_elec” component is +0.315 REUs. **B**: The protein PDB ID is 3IN7, the ligand ZINC ID is ZINC00792906. This decoy complex is ranked 3rd by score12 and 16th by Talaris, with interface energies of -8.706 and -7.288 REUs, respectively. Here the solvation component accounts for 72% of the difference in interface energy, its value being +10.147 REUs under score12 and +11.172 REUs under Talaris. Another 26% of the difference in interface energy is contributed by the hydrogen-bond component; in particular, the hydrogen bonds between the ligand and the protein (yellow broken lines) have a total energy of -0.925 REUs under score12 and -0.558 REUs under Talaris.
(TIFF)

S2 Fig Rationalization of a decoy that ranks favorably with EEF1’s polar solvation, but less so with pwSHO’s polar solvation. The protein PDB ID is 4G55 and the ligand ZINC ID is ZINC00624571. To appropriately compare between the two energy functions, the same conformation was scored using both energy functions (i.e. without minimization). This decoy complex is ranked 10th by Talaris+EEF1 and 32nd by Talaris+pwSHO, with protein-ligand interface energies of -6.611 and -6.369 REUs, respectively. The entire difference in interface energy is contributed by the polar solvation component, whose EEF1 value is +8.721 REUs and whose pwSHO value is +8.964 REUs. To help understand why pwSHO penalizes this complex more than EEF1, we present the interaction between the protein backbone carbonyl atom O(65) and atom S1 of the ligand. The energetic cost for desolvating O(65) with S1 is +0.05 REUs under EEF1, whereas it is +0.10 REUs under pwSHO. The same applies for the interaction of the protein sidechain arginine atom N^η2^(64) with atom C23 in the ligand. The energetic cost for desolvating N^η2^(64) with C23 is +0.12 REUs under EEF1, versus +0.22 REUs under pwSHO. In both of these cases, the pwSHO energy more strongly penalizes the fact that ligand binding reduces this ability of these protein atoms to hydrogen bond with nearby water. This image was produced using PyMOL [47], with the protein represented in spheres and the ligand represented in sticks.
(TIFF)

S3 Fig Identification of the known inhibitor using a FRED-docked model of the complex. Scoring in this experiment was carried out using the Talaris+pwSHO energy function. **(A)** Performance for this task is diminished significantly (p = 0.019) if the known inhibitor is docked, rather than provided in the experimentally-determined pose: with 3 versus 11 “wins”, respectively. **(B)** The fold-decrease in performance observed upon using the docked structure of the known inhibitor, as opposed to the experimentally determined structure, appears uncorrelated to the RMSD between the two structures. Performance decreases even in some of the cases for which the RMSD of the FRED-docked structure is less than 2 Å from the native pose, suggesting that even this accuracy may be insufficient for the known inhibitor to be selected by the downstream scoring function.
(EPS)

S4 Fig Identification of the known inhibitor using its “default” tautomeric state and protonation state. Performance for this task is diminished significantly (p = 0.0008) if the tautomeric state and protonation state of the known inhibitor are computed with QUACPAC (i.e. without knowledge of the protein environment) rather than with Protoss (i.e. using the experimentally-derived structure of the native complex). There are zero “wins” using the QUACPAC ligand configuration, versus ten “wins” using the Protoss ligand configuration. Scoring in this experiment was carried out using the Talaris+pwSHO energy function.
(EPS)

S5 Fig Identification of the known inhibitor using non-minimized complexes. Scoring in this experiment was carried out using the Talaris+pwSHO energy function. **(A)** Performance for this task becomes a much easier if performed on non-minimized rather than minimized complexes, due to the presence of steric clashes in non-minimized decoy complexes. The increase in performance is dramatic (p = 0.000004), with the known inhibitor being ranked 10 times as first overall, and 15 times in the top 1% of the compound library. **(B)** The fold-increase in performance upon using non-minimized complexes, as opposed to minimized complexes, appears uncorrelated with the RMSD between minimized and non-minimized structures of the known inhibitor. This is consistent with the expectation that minimization makes the decoy complexes more competitive, and thus the change in performance is unrelated to how minimization affects the active complex. RMSD was computed over the inhibitor’s heavy atoms, after superposition of the protein residues within 10 Å of the inhibitor.
(EPS)

S6 Fig Rationalization of cases where FRED Chemgauss4 and Talaris+pwSHO rank the known inhibitor discordantly. To appropriately compare between the two energy functions, the same conformation was scored using both energy functions (i.e. without minimization). Both images were produced using PyMOL [47], with the protein represented in spheres and the ligand represented in sticks. **A**: The PDB ID of this active complex is 4GQ4. The known inhibitor is ranked 3rd by FRED Chemgauss4, but 23rd by Talaris+pwSHO. Both scoring functions agree that the inhibitor has moderately favorable hydrogen-bonding and polar-solvation energies. A prominent difference between the two scoring functions is in the evaluation of non-polar solvation: while Chemgauss4 has no explicit term to model non-polar solvation, Talaris+pwSHO ranks the inhibitor's “lk_nonpolar” energy term very unfavorably (2461st out of 2501). **B**: The PDB ID of this active complex is 2YEL. The known inhibitor is ranked 1st by Talaris+pwSHO, but 371st by Chemgauss4. Under Chemgauss4 the inhibitor has unfavorable shape complementarity (ranking 682nd by combined “CG3:Steric” and “CG3:Clash” scores) and unfavorable intermolecular hydrogen bonding (434th by “CG4:HB” score). By contrast, under Talaris+pwSHO the inhibitor has excellent shape complementarity (3rd by combined “fa_atr” and “fa_rep” energies), and favorable hydrogen bonding (42nd by combined “hb_bb_sc” and “hb_sc” energies).
(TIFF)
