# Supplementary figures and images for: Enhancements to the Rosetta Energy Function Enable Improved Identification of Small Molecules that Inhibit Protein-Protein Interactions
Source: PLoS One. 2015 Oct 20;10(10):e0140359. doi: 10.1371/journal.pone.0140359 (PMC4617380; doi:10.1371/journal.pone.0140359)

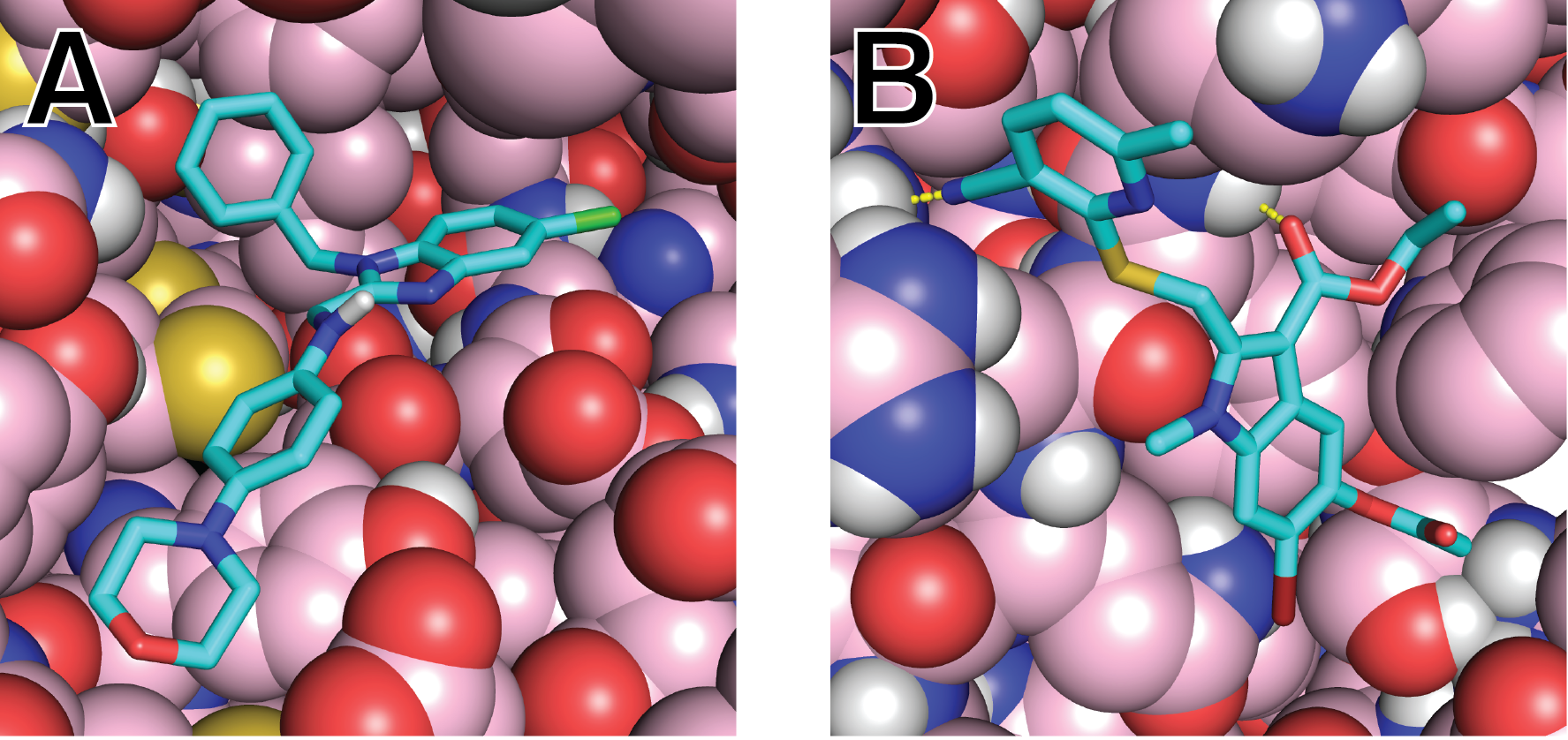

Supplement: S1 Fig — To appropriately compare between the two energy functions, the same conformation was scored using both energy functions (i.e. without minimization). Both images were produced using PyMOL [47], with the protein represented in spheres and the ligand represented in sticks. (A) The protein PDB ID is 4GQ4, the ligand ZINC ID is ZINC01019586. This decoy complex is ranked 10th by score12 and 34th by Talaris, with protein-ligand interface energies of -10.522 and -8.913 Rosetta Energy Units (REUs), respectively. Seventy-three percent of the difference in interface energy is due to the solvation component of the interface energy, which is +8.397 under score12 and +9.564 under Talaris. The change reflects the increase from 0.65 in score12 to 0.75 in Talaris in the weight that solvation energy has in the computation of total energy; the unweighted solvation components are indeed very similar: +12.918 for score12 and +12.752 for Talaris. The electrostatic component contributes 20% of the difference in interface energy, because the score12 “fa_pair” component is 0 REUs, whereas the Talaris “fa_elec” component is +0.315 REUs. B: The protein PDB ID is 3IN7, the ligand ZINC ID is ZINC00792906. This decoy complex is ranked 3rd by score12 and 16th by Talaris, with interface energies of -8.706 and -7.288 REUs, respectively. Here the solvation component accounts for 72% of the difference in interface energy, its value being +10.147 REUs under score12 and +11.172 REUs under Talaris. Another 26% of the difference in interface energy is contributed by the hydrogen-bond component; in particular, the hydrogen bonds between the ligand and the protein (yellow broken lines) have a total energy of -0.925 REUs under score12 and -0.558 REUs under Talaris. (TIFF) [file pone.0140359.s004.tiff]

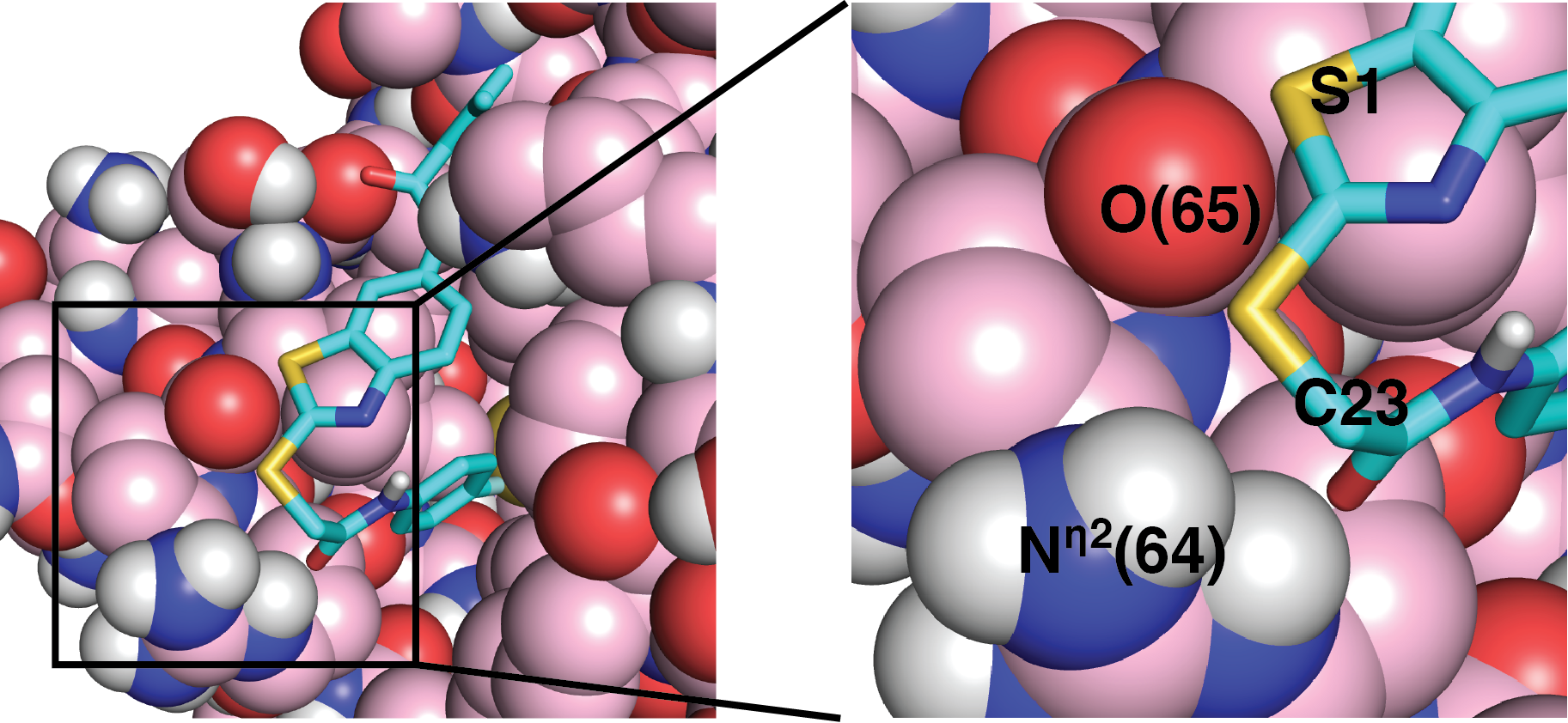

Supplement: S2 Fig — The protein PDB ID is 4G55 and the ligand ZINC ID is ZINC00624571. To appropriately compare between the two energy functions, the same conformation was scored using both energy functions (i.e. without minimization). This decoy complex is ranked 10th by Talaris+EEF1 and 32nd by Talaris+pwSHO, with protein-ligand interface energies of -6.611 and -6.369 REUs, respectively. The entire difference in interface energy is contributed by the polar solvation component, whose EEF1 value is +8.721 REUs and whose pwSHO value is +8.964 REUs. To help understand why pwSHO penalizes this complex more than EEF1, we present the interaction between the protein backbone carbonyl atom O(65) and atom S1 of the ligand. The energetic cost for desolvating O(65) with S1 is +0.05 REUs under EEF1, whereas it is +0.10 REUs under pwSHO. The same applies for the interaction of the protein sidechain arginine atom Nη2(64) with atom C23 in the ligand. The energetic cost for desolvating Nη2(64) with C23 is +0.12 REUs under EEF1, versus +0.22 REUs under pwSHO. In both of these cases, the pwSHO energy more strongly penalizes the fact that ligand binding reduces this ability of these protein atoms to hydrogen bond with nearby water. This image was produced using PyMOL [47], with the protein represented in spheres and the ligand represented in sticks. (TIFF) [file pone.0140359.s005.tiff]

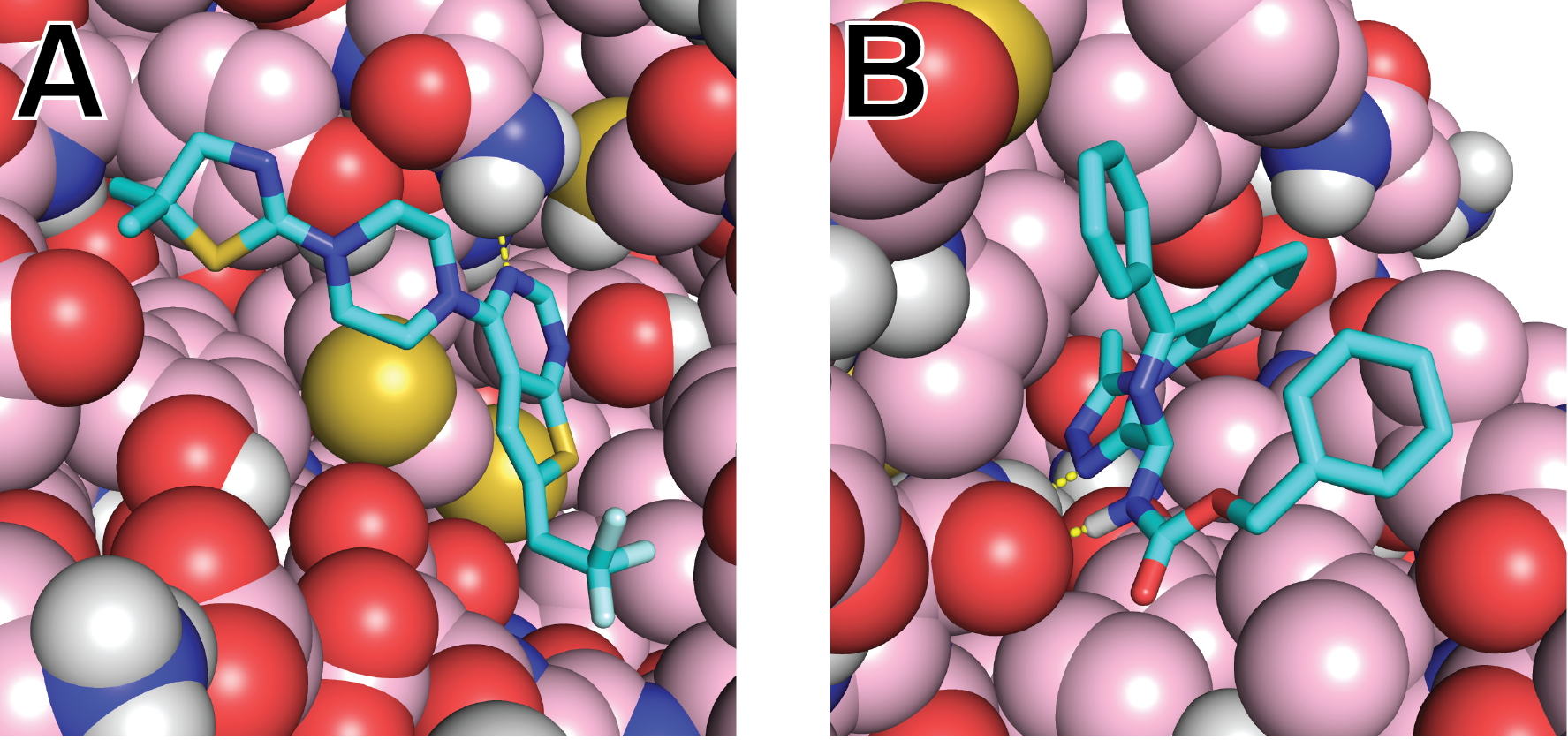

Supplement: S6 Fig — To appropriately compare between the two energy functions, the same conformation was scored using both energy functions (i.e. without minimization). Both images were produced using PyMOL [47], with the protein represented in spheres and the ligand represented in sticks. A: The PDB ID of this active complex is 4GQ4. The known inhibitor is ranked 3rd by FRED Chemgauss4, but 23rd by Talaris+pwSHO. Both scoring functions agree that the inhibitor has moderately favorable hydrogen-bonding and polar-solvation energies. A prominent difference between the two scoring functions is in the evaluation of non-polar solvation: while Chemgauss4 has no explicit term to model non-polar solvation, Talaris+pwSHO ranks the inhibitor's “lk_nonpolar” energy term very unfavorably (2461st out of 2501). B: The PDB ID of this active complex is 2YEL. The known inhibitor is ranked 1st by Talaris+pwSHO, but 371st by Chemgauss4. Under Chemgauss4 the inhibitor has unfavorable shape complementarity (ranking 682nd by combined “CG3:Steric” and “CG3:Clash” scores) and unfavorable intermolecular hydrogen bonding (434th by “CG4:HB” score). By contrast, under Talaris+pwSHO the inhibitor has excellent shape complementarity (3rd by combined “fa_atr” and “fa_rep” energies), and favorable hydrogen bonding (42nd by combined “hb_bb_sc” and “hb_sc” energies). (TIFF) [file pone.0140359.s009.tiff]
